# The consequences of the pandemic among patients with psychiatric history

**DOI:** 10.1192/j.eurpsy.2022.1389

**Published:** 2022-09-01

**Authors:** A.V. Gurita, A.H.I. Abu Shehab, L. Luca, N. Isabela, M. Terpan, A. Ciubara

**Affiliations:** 1 ’Elisabeta Doamna’’ Psychiatry Hospital of Galati, Psychiatry Department, Galati, Romania; 2 ’’Elisabeta Doamna’’ Psychiatry Hospital of Galati, Psychiatry Department, Galati, Romania; 3 University of Medicine and Pharmacy “Grigore T. Popa”, Psychology, Iasi, Romania; 4 ’Dunerea de jos’’ University of Galati, Psychiatry Department, Galati, Romania

**Keywords:** mental health, psychiatry, pandemic

## Abstract

**Introduction:**

Throughout this period we were confronted with news and information about the Corona virus and its consequences. Which led to the development of a huge sense of fear among people. Although fear has helped to maintain restrictions, it has also had a significant impact on mental health, especially among patients with a psychiatric history.

**Objectives:**

In this paper I will highlight the consequences of the nocebo effect of the pandemic among people with a psychiatric history.

**Methods:**

To complete this work I used medical articles, studies, and specialized information on the subject.

**Results:**

The pandemic’s restrictions have made it difficult for psychiatric patients to be compliant treatment by avoiding regular psychiatric exams.Isolation and fear of infection has led to new decompensations in existing psychiatric pathologies.

**Conclusions:**

The exacerbations of psychiatric pathology increased both in number and in their intensity, ultimately determined by the increase in the number of hospitalizations in psychiatric emergencies.

**Disclosure:**

No significant relationships.

